# Acute Effects of Caffeinated Chewing Gum on Volleyball Performance in High-Performance Female Players

**DOI:** 10.2478/hukin-2022-0092

**Published:** 2022-11-08

**Authors:** Aleksandra Filip-Stachnik, Magdalena Kaszuba, Bartlomiej Dorozynski, Zuzanna Komarek, Dawid Gawel, Juan Del Coso, Tomasz Klocek, Michal Spieszny, Michal Krzysztofik

**Affiliations:** 1 Institute of Sport Sciences, The Jerzy Kukuczka Academy of Physical Education in Katowice, Katowice, Poland.; 2 Nutrition and Sports Performance Research Group, The Jerzy Kukuczka Academy of Physical Education in Katowice, Katowice, Poland.; 3 Centre for Sport Studies, Rey Juan Carlos University, Fuenlabrada, Spain.; 4 Institute of Sports Sciences, University of Physical Education in Krakow, Krakow, Poland.

**Keywords:** caffeine, sports performance, team sports, sports nutrition, supplement

## Abstract

To date, no investigation has studied the effect of acute intake of caffeinated chewing gum on volleyball performance. Therefore, the aim of this investigation was to establish the impact of caffeinated chewing gum ingestion on physical performance in female volleyball players. Twelve high-performance volleyball female athletes participated in a randomized, crossover, placebo-controlled, and double-blind experiment. Each athlete performed two identical experimental sessions after a) ingestion of ~6.4 mg/kg of caffeine via caffeinated chewing gum, b) ingestion of non-caffeinated chewing gum as a placebo. After the ingestion of gum, athletes performed a volleyball game, and performance was assessed by a notational analysis. Just before and after the game, jump performance during block and attack actions was evaluated. The number of points obtained and the number of errors committed during serve, reception, attacking, and blocking actions were unaffected by the ingestion of caffeinated chewing gum (p from 0.066 to 0.890). However, caffeinated chewing gum increased jump attack height in comparison to the placebo (pre-game 46.0 ± 7.2 vs. 47.2 ± 6.7 cm, p = 0.032; post-game 46.3 ± 7.6 vs. 47.5 ± 6.9 cm, p = 0.022, respectively). Caffeinated chewing gum did not modify block jump height (pre-game 32.7 ± 5.5 and 33.0 ± 4.3 cm, p = 0.829; post-game: 34.8 ± 6.1, 35.4 ± 6.1 cm, p = 0.993, respectively). The ingestion of ~6.4 mg/kg of caffeine via caffeinated chewing gum was effective for improving jump attack performance in women volleyball athletes. However, this effect was not translated into better volleyball performance during a game.

## Introduction

Volleyball is an intense team sport characterized by short duration periods (i.e., 3–9 s) at high intensity during gameplay, interspersed with periods of recovery (García-de-Alcaraz et al., 2020) during the pauses between points. Although actions performed by volleyball players may differ in terms of their playing position, common movements include accelerations and decelerations, jumping, ball-spiking, and multidirectional locomotion (Sheppard et al., 2007). Among them, jump performance has been reported as the most essential physical attribute for defensive and offensive actions (Ziv and Lidor, 2010). Indeed, most actions (i.e., spike, block, serve, and setting) are performed while jumping vertically and have been directly related to game success (Sheppard et al., 2007).

In volleyball, the use of caffeine in doses between 3 and 6 mg/kg has been found effective in increasing countermovement jump (Coso et al., 2014; Pérez-López et al., 2015; Zbinden-Foncea et al., 2018), squat jump (Coso et al., 2014; Pérez-López et al., 2015), attack and block jump performance (Pérez-López et al., 2015), although this is not always the case (Fernández-Campos et al., 2015). Unfortunately, only one study (Pérez-López et al., 2015) explored the acute effects of caffeine using volleyball-specific jumping procedures (i.e., attack and block jump) despite those specific movement patterns have been indicated as absolutely crucial when testing high-performance volleyball athletes (Sattler et al., 2012). Moreover, in all previous investigations, jumping capacity was assessed only in a non-fatigue state, which may not accurately reflect in-game, sport-specific jumping tasks. Considering that each athlete may perform more than 250 jumps during a match (Stanganelli et al., 2008), maintaining the ability to jump across the match may be a key element for volleyball performance. Therefore, evaluating the effects of caffeine on jump performance across the match seems justified to elucidate the true potential benefit of pre-exercise caffeine intake in volleyball.

Previous investigations that explored caffeine’s effects on volleyball performance used caffeine in the anhydrous form, administered in gelatin capsules (Zbinden-Foncea et al., 2018) or energy drinks (Coso et al., 2014; Fernández-Campos et al., 2015; Pérez-López et al., 2015), consumed 45-60 min before the onset of testing. An alternative caffeine delivery method is caffeinated chewing gum. This form of caffeine supplementation was analyzed in other sports disciplines and showed positive (Dittrich et al., 2019; Paton et al., 2015) and neutral effects (Evans et al., 2018; Filip-Stachnik, Krawczyk, et al., 2021). Since caffeinated chewing gum allows absorption of caffeine directly into the bloodstream via the buccal mucosa (thereby bypassing hepatic metabolism), 85% of the caffeine administered in the gum can be bioavailable after 5 min of chewing (Kamimori et al., 2002). Thus, using caffeinated chewing gum instead of caffeine capsules may be of value when fast absorption of caffeine is desirable. Given that the duration of a volleyball match may differ significantly depending on the final sets (3 to 5 sets; 1 to 3 hours), the use of caffeinated chewing gum may be an interesting supplementation protocol just prior to or during the match. However, the potential benefits of using caffeine intake via chewing gum has not been previously established for volleyball performance. The aim of this investigation was to determine the acute effects of 6 mg/kg of caffeine via chewing gum on volleyball performance. Based on the results of previous studies on volleyball (Coso et al., 2014; Pérez-López et al., 2015; Zbinden-Foncea et al., 2018) and regarding diminished fatigue properties of caffeine (Graham, 2001), we hypothesized that the intake of caffeinated chewing gum would enhance volleyball-specific performance compared to the placebo/non-caffeinated gum.

## Methods

### Participants

To calculate the number of participants to be included in the investigation, statistical software G*Power (Dusseldorf, Germany) was used, including the following variables in the *a priori* calculation of the required sample size: the ANOVA, repeated measures, within factors was assumed as a statistical test to be employed, a large effect size of caffeine (ES) = 1.22 was expected (Fernández-Campos et al., 2015), an alpha value of 0.05, a statistical power of 80%, and a correlation between measurements of 0.50. All this information was introduced considering that the current experiment was a cross over study, and one group of athletes would perform two experimental conditions (i.e., caffeinated chewing gum vs. non-caffeinated chewing gum). Power analysis indicated that a sample of at least 5 athletes were required for this study. Considering that the procedure assumed testing during a volleyball game, we recruited 12 female volleyball players to participate in the study. Due to a knee injury during jumping in the past, one participant (libero) refused to join in the jumping part of the experiment, yet took part in the remaining game assessments. The main characteristics of the study sample are depicted in Table 1. Athletes were recruited from the same volleyball team (2^nd^ division of the Polish National league), and testing was conducted immediately after the competitive season. The inclusion criteria were as follows: (a) free from neuromuscular and musculoskeletal disorders; (b) no medication nor dietary supplements used within the previous month, which could potentially affect the study outcomes (e.g., beta-alanine, creatine, etc.); (c) self-described good health status. Athletes were excluded if they reported a) positive smoking status; b) potential allergy to caffeine. Six participants were tested during the follicular phase of their menstrual cycle, whereas the remaining six athletes were tested during the luteal phase. Previous investigations suggested that the response to caffeine intake in resistance exercise (Romero-Moraleda et al., 2019) and anaerobic-like exercise (Lara et al., 2020) is similar across the menstrual cycle; thus, all participants were treated as a single group. Habitual caffeine intake was measured using a modified version of the validated questionnaire by Bühler et al. (2014) that recorded the type and amount of caffeine-containing foods and dietary supplements and was assessed for the four weeks before the experiment (Filip et al., 2020). According to previous recommendations, athletes were classified as mild caffeine users (Table 1) (Filip et al., 2020). The study protocol was approved by the Academy Ethics Committee (3/2019) per the latest version of the Declaration of Helsinki. All athletes provided their written informed consent before participation in this study.

**Table 1:** Main participants’ characteristics.

Variable [units]	Value (mean ± SD)
Age [years]	20 ± 2
Body mass [kg]	69.1 ± 2.3
Body height [cm]	178 ± 6
Volleyball training experience [years]	10 ± 2
Habitual caffeine intake [mg/kg/day]	2.7 ± 2.1

### Pre-experimental standardizations

Before the first experimental trial, athletes were instructed to maintain their usual hydration and dietary habits (including pre-workout meals) and habitual caffeine intake during the study period. To produce a within-subject standardization of diet, athletes replicated the same nutritional pattern before each trial, which was verified by a qualified nutritionist. Athletes were also asked to refrain from any source of caffeine and alcohol 24 h before each experimental trial and not to perform strenuous exercise within 24 h before testing.

### Experimental design

To investigate the effect of caffeinated chewing gum intake on volleyball performance, athletes participated in a randomized, double-blind, counterbalanced, placebo-controlled crossover experiment where each athlete acted as their control. The randomization was performed by a research team member who was not involved in the data collection; thus, athletes and researchers were blinded to the trials. Each athlete took part in one familiarization session and then two identical experimental trials that included ingesting chewing gum 15 min before testing. In the experimental trials, chewing gum contained (a) ~6.4 ± 0.2 mg/kg of caffeine (range: 6.0 – 6.6 mg/kg) or (b) an inert substance to produce a placebo (i.e., non-caffeinated chewing gum). Caffeine was administered as an absolute dose of 400 mg for all participants using four pieces of commercially available caffeinated chewing gum (Military Energy Gum; MarketRight Inc., Plano, IL, USA). An absolute dose of caffeine was used instead of a relative dose as the preparation of individual chewing gum was unfeasible. Placebo gum was a commercially available non-caffeinated chewing gum with a similar taste, color and shape (Airways, Poland). The experimental trials were separated by 72 h to allow complete recovery and substance wash-out. On the experimental days, participants arrived at their habitual training time at 4 PM to the gym. Each participant received an opaque container that contained caffeinated chewing gum or placebo gum cut into small pieces upon arrival. Athletes chewed gum continuously for 5 min and were required to expectorate chewed gum into a container. Next, athletes conducted a standardized habitual pre-workout warm-up lasting approximately 15 min. The warm-up consisted of 5 min of continuous running, followed by dynamic stretching exercises for the upper and lower limbs and specific volleyball exercises (Krzysztofik et al., 2021). Next, players performed a volleyball jump performance test that included baseline attack and block jump height assessments, a volleyball game and a repetition of the attack and block jump height assessments. Each team included players who ingested caffeinated and placebo gum during the game. Heart rate during the game was measured using a heart rate monitor (Polar H10, Finland). After finishing testing, athletes were asked about occurring side effects connected with caffeine ingestion and their perception of performance during testing.

### Jumping assessment

After the warm-up, athletes performed two attack jumps. During the attack jump, athletes used an individually determined 2- to 3-step approach, performing a bounce jump with an arm swing. Athletes were instructed to jump as high as possible by completing the jumping procedure in the way that they found most convenient, similar to their habitual technique, but without a ball (Sattler et al., 2012). Then, players performed two-block jumps. The block jump was performed in a defensive volleyball position simulating actions performed during a match. The hands were positioned in front of the chest, and then, participants performed a countermovement jumping-type technique with self-determined countermovement depth and the amount of arm swing typical for their individual volleyball technique during a game. Players performed the vertical jump with full arm extension to reach as high as possible (Sattler et al., 2012). Jumps were separated by a 1-min rest interval and were conducted within 5 min from completion of the match. The best jump of the two attempts for each jump type was used for further analysis. Jump height was measured using the Optojump photoelectric cells (Microgate, Bolzano, Italy). This device has been previously proven as valid and reliable for assessing vertical jump performance (Glatthorn et al., 2011).

### Game assessment

After at least 5 min of rest, athletes participated in a volleyball game on an official volleyball court. Athletes played one set according to the rules of the International Volleyball Federation. The team’s coach acted as a referee to decide on play disputes during the game. The game was recorded using two synchronized video cameras (Sony FDR191 AX53) set diagonally (approximately 10 m at the back of each half-court and placed 2 m above the floor) to record each game action during the simulated game. To avoid the effects of the rival’s level on the results of this study, the two teams competing were composed of the same six players for both experimental trials, and played at their specific positions. Two experienced observers collected the game data using Data Volley software (DataProject, Bologna, Italy). Specifically, quantitative statistics for each player were obtained during serve, reception, attack and block actions (Silva et al., 2014) (Table 2). Observers that introduced the data were blinded to the experimental treatments. The number of jumps performed by every athlete during the game was also collected.

**Table 2:** Cluster classification of volleyball game actions during a simulated game.

Action	Description
Service error	Net ball, out, foot foul
Service point	Direct point from a service (the opponent can’t receive or loses the ball)
Reception error	Direct point for the opponent from a serve
Negative reception	Reception that ends 3 or more meters from the net and the setter is not able to prepare a quick/clear spike or the ball goes into the opponent field, but is not attacked directly and/or successfully
Positive reception	Ball received within the 3-meter line which allows the setter to prepare more than one attack
Perfect reception	Reception through which the ball reaches an optimal setting zone with a perfect parabola, allowing the setter to prepare all the attack options
Attack error	A spike ends in the net, out or a player touched the net during the attack
Attack point	Direct point or side-out gained during a spike action
Blocking point	The defenders obtain a direct point from a blocking action of the opponents’ attack
Total points	The sum of all the points obtained in the game
Total errors	The sum of all the errors obtained in the game

All data are presented as mean ± standard deviation for a group of high-performance female volleyball players.

### Side effects and assessment of blinding

Immediately after finishing testing, athletes were asked about their feelings associated with typical caffeine-induced side effects. Additionally, athletes were asked about their perception of performance during testing (Filip-Stachnik, Krzysztofik, et al., 2021b). The effectiveness of blinding was explored pre- and post-exercise by asking participants the following question: Which supplement do you think you have ingested?. This question had three possible responses: (a) “caffeine”, (b) “placebo”, and (c) “I do not know”.

### Statistical analysis

All calculations were performed using Statistica 13.3 and expressed as means with standard deviations (±SD) for the whole participants. According to the Shapiro-Wilk test, the jump attack and block height, and the mean and peak heart rate presented a normal distribution. A two-way ANOVA with repeated measures (2 condition × 2 time) was used to evaluate the effects of caffeine administration during the attack and block jumps. In the event of a significant main effect, post-hoc comparisons were conducted using the Tukey’s test. Differences in mean and peak heart rate were determined using paired T-tests. Differences in the variables during the simulated game were determined by paired T-tests or Wilcoxon's tests according to their distribution. Differences in perception of performance during testing were determined using a Pearson’s chi-squared test (x^2^). Effect sizes (ES) were calculated using Hedges *g* for repeated measures. ES of 0.00–0.19, 0.20–0.49, 0.50–0.79, and ≥ 0.80 represented trivial, small, moderate, and large effects, respectively (Hedges 1981). Statistical significance was set at *p* < 0.05. The effectiveness of blinding was examined using the Bang’s Blinding Index (BBI). Analyses of the BBI were performed using R Statistical Software V 3.6.3 (RStudio Team, 2019) applying the add-on package 'BI'.

## Results

### Jumping performance assessment

The results of the two-way repeated measures ANOVA showed a significant main effect for condition in attack jump height (F_(1,10)_= 7.05; *p* = 0.024), without significant main effect for time (F_(1,10)_ = 0.26, *p* = 0.622) and condition × time interaction effect (F_(1,10)_ = 0.03; *p* = 0.869). The pairwise comparisons revealed a significant improvement between the placebo and the caffeine trial in jump attack height in pre-game and post-game measurements (*p* = 0.032, *p* = 0.022, respectively; Table 3).

**Table 3:** Attack and block jump height before (PRE) and after (POST) a volleyball game with the ingestion of caffeinated chewing gum (~6.4 mg/kg of caffeine) and non-caffeinated chewing gum (placebo).

Time	Placebo	Caffeine	Placebo vs. Caffeine ES
Attack Jump [cm]			
PRE	46.0 ± 7.9	47.2 ± 7.3*	0.15
POST	46.3 ± 8.3	47.5 ± 7.5*	0.15
Block Jump [cm]			
PRE	32.6 ± 5.7	33.0 ± 4.5	0.07
POST	34.8 ± 6.4#	34.7 ± 6.2#	0.02

PRE: pre-game; POST: post-game. ES: effect size. CI: confidence interval. *Statistically significant difference (p < 0.05) in comparison to the placebo. # Statistically significant difference (p < 0.05) in comparison to PRE. All data are presented as mean ± standard deviation for a group of high-performance female volleyball players.

The results of two-way repeated measures ANOVA showed a significant main effect of time in block jump height (F_(1,10)_ = 11.36; *p* = 0.007), without main effect for condition (F_(1,10)_ = 0.13, *p* = 0.724) and condition × time interaction effect (F_(1,10)_ = 0.63; *p* = 0.448). Pairwise comparisons revealed a significant improvement in block jump height after the game for both the placebo and caffeine trials (Table 3; *p* < 0.050).

### Game assessment

The results of paired T-tests indicated non-significant differences between the placebo and the caffeine trial in mean (*p* = 0.724; 136 ± 10 and 134 ± 12 bpm, respectively), as well as peak heart rate (*p* = 0.794; 178 ± 11 and 176 ± 10 bpm, respectively) during the volleyball game. Additionally, no significant differences determined by paired T-tests were found in the number of jumps performed during placebo and caffeine trials (*p* = 0.273; 47 ± 15 and 52 ± 13 jumps, respectively).

The results of paired T tests showed no significant differences in total points, total errors, service points, service errors, attack points and attack errors between conditions (*p* = 0.601, *p* = 0.723, *p* = 0.339, *p* = 0.082, *p* = 0.551, *p* = 0.006, respectively; Table 3). Results of the Wilcoxon’s test revealed no significant differences in the reception errors, negative reception, positive reception, perfect reception, and blocking points (*p* = 0.102, *p* = 0.890, *p* = 0.786, *p* = 0.180, *p* = 0.480, respectively; Table 4) between conditions.

**Table 4:** Game-related statistics during a simulated volleyball game with the ingestion of caffeinated chewing gum (~6.4 mg/kg of caffeine) or non-caffeinated chewing gum (placebo).

Game related statistics (number)	Placebo	Caffeine	*p value*	*Placebo vs. Caffeine* ES
Serve error	0.2 ± 0.4	0.4 ± 0.7	0.082	0.46
Serve point	0.4 ± 0.7	0.3 ± 0.5	0.339	0.30
Reception error	0.6 ± 1.0	0.3 ± 0.6	0.102	0.40
Negative reception	0.9 ± 1.5	0.9 ±1.2	0.890	0.00
Good reception	1.0 ± 1.8	1.1 ± 1.6	0.786	0.05
Perfect reception	0.2 ± 0.4	0.4 ± 0.7	0.180	0.46
Attack error	1.2 ± 1.3	1.2 ± 1.3	0.066	0.00
Attack point	3.9 ± 3.7	4.4 ± 4.3	0.551	0.12
Blocking point	0.8 ± 1.0	1.0 ± 1.0	0.408	0.26
Total points	5.1 ± 3.9	5.7 ± 5.0	0.601	0.13
Total errors	1.9 ± 1.5	1.8 ± 1.6	0.723	0.06

All data are presented as mean ± standard deviation for a group of high-performance female volleyball players.

### Side effects and assessment of blinding

During the placebo condition, no athlete reported any side effects. During the caffeine trial, one athlete reported hand tremors. One athlete reported problems with chewing gum in both conditions because she was using a permanent retainer. The Chi-square test did not reveal any difference for the self-perception of performance improvement between conditions (*p* = 0.098; 25% and 58% of “yes” responses for placebo and caffeine trials, respectively). In the pre-exercise assessment, 58% and 67% of athletes correctly identified the placebo and caffeine conditions, respectively. In the post-exercise evaluation, 67% of athletes correctly identified the placebo and caffeine conditions (Table 5).

**Table 5:** Guessed responses before and after a simulated volleyball match with the ingestion of caffeinated chewing gum (~6.4 mg/kg of caffeine) or non-caffeinated chewing gum (placebo).

Condition	Responded as the placebo	Responded as caffeine	Responded as “I don't know”	Bang’s Blinding Index
		Pre-exercise		
Placebo	7	4	1	0.42
Caffeine	3	8	1	0.25
		Post-exercise		
Placebo	8	3	1	0.42
Caffeine	3	8	1	0.42

Data represent the number of participants in each response option in a group of high-performance female volleyball players.

## Discussion

The main purpose of this study was to investigate the acute effects of ~6 mg/kg of caffeine ingestion via chewing gum on volleyball performance before, during and after a simulated game in high-performance female volleyball players. The results of the present study indicate that the ingestion of ~6 mg/kg of caffeine via chewing gum significantly improved attack jump height before and after a simulated game. However, caffeinated chewing gum had no performance benefits to block jump height, nor to any of the performance variables measured during the simulated game, such as the number of points and errors during serve, reception, attack and block actions. Collectively, these outcomes suggest that ingestion of ~6 mg/kg of caffeine via chewing gum was effective to obtain an improvement in attack jump performance with no changes during the block jump. This information may suggest that caffeine can be more effective in increasing jump height in volleyball-specific jump actions that require an approach and arm swinging. However, the ergogenic benefit of using caffeinated chewing gum to attack jump performance was not translated into better volleyball performance during a simulated game.

Several previous studies have analyzed the effectiveness of acute caffeine intake on jumping performance in team sports (Del Coso et al., 2014; Del Coso, Muñoz-Fernández, et al., 2012; Pérez-López et al., 2015; Puente et al., 2017; Ranchordas et al., 2018; Zbinden-Foncea et al., 2018). In those investigations caffeine improved countermovement jump performance in basketball (Abian et al., 2015), volleyball (Del Coso et al., 2014; Pérez-López et al., 2015; Zbinden-Foncea et al., 2018), soccer (Ranchordas et al., 2018) and futsal (López-Samanes et al., 2021) players, which is partially in line with the results of current investigation. Interestingly, the results of present research show that caffeine significantly improved attack jump height (pre-and post-game: +2.6% for both) without a significant impact on block jump height. It is worth noticing that the differences between the results in the two jumps investigated in the current study may be explained by the technique used during those specific volleyball movements (Sattler et al., 2012). During the block jump, athletes start with the knees slightly bent and arms in front of the chest to perform a jump with no full arm swing. In the study by Sattler et al. (2012), authors indicated that during block jumps, athletes used a somewhat shortened version of the countermovement jump technique. On the other hand, during the attack jump, athletes used a 2- to 3-step approach, performing a half-drop jump followed by a countermovement jump with an arm swing and eccentric contraction. Therefore, the attack jump exploits the stretch-shortening cycle of the engaged muscles to a greater extent (Sattler et al., 2012). Since previous studies suggest that caffeine improves the stretch-shortening cycle (Wilk et al., 2019), the possible differences might depend on its more effective utilization during attack jump after caffeine intake. In light of the current results and previous investigations, it seems adequate to suggest that the ingestion of caffeine enhances countermovement jump performance in volleyball players.

Numerous previous studies have explored the impact of caffeine intake on single (Del Coso et al., 2014; Puente et al., 2017; Ranchordas et al., 2018; Zbinden-Foncea et al., 2018) and repeated jumping performance in team sports (Abian et al., 2015; Del Coso et al., 2014; Del Coso, Salinero, et al., 2012; Ribeiro et al., 2016). However, to the best of our knowledge, this is the first study that assessed the effects of acute caffeine intake on jumping ability before and after a volleyball game. This may be especially significant in volleyball, where almost all technical skills during the match (i.e., serving, spiking, blocking and setting) are performed while jumping. Thus, maintaining jump performance throughout the whole game seems crucial for success in volleyball (Sattler et al., 2012). Results of the present study show that ingestion of ~6 mg/kg of caffeine via chewing gum improved the jumping capacity before the simulated volleyball game and the ergogenic benefit was still present with the same magnitude after the game. Thus, results of the current investigation confirm the ability to maintain jumping performance during exercise and complement the outcomes observed during repeated sprints (Del Coso, Salinero, et al., 2012; Evans et al., 2018; Kopec et al., 2016), repeated jumps (Abian et al., 2015; Del Coso et al., 2014; Del Coso, Salinero, et al., 2012; Ribeiro et al., 2016), the Yo-Yo test (Ranchordas et al., 2018) and in the distance covered during the match (Del Coso, Salinero, et al., 2012; Lara et al., 2014) in team sports.

It should be taken into account that team sport athletes require an appropriate level of different motor abilities, as well as other sport-specific technical and tactical skills (Salinero et al., 2019). Previous studies have shown that caffeine improved positive actions (Del Coso et al., 2014; Pérez-López et al., 2015) and reduced neutral (Del Coso et al., 2014) and negative (Pérez-López et al., 2015) ones, which was confirmed by game analysis of experienced volleyball coaches, yet it was not observed in the current investigation. The source of caffeine used in those investigations in comparison to the current study (energy drink vs. gum) could be a possible explanation of differences in the obtained results. It is possible that the use of chewing gum to deliver a given dose of caffeine is less effective than a capsule with caffeine or a caffeinated energy drink. This is because the whole amount of caffeine is ingested orally with the capsule or the drink while caffeine is progressively liberated from the gum during the chewing action (Kamimori et al., 2002). It thus seems possible that part of the caffeine remains in the gum after 5 min of chewing. Interestingly, several previous investigations have found that the use of caffeinated chewing gum may not be totally effective to increase physical performance in several sport situations (Filip-Stachnik, Krawczyk, et al., 2021; Russell et al., 2020; Ryan et al., 2013). Further investigations comparing various sources of caffeine in different doses are needed to confirm this statement.

Since caffeine ingestion can provoke side effects, including nausea and anxiety (Graham, 2001), this may negatively impact volleyball performance during competition. Interestingly, a very low prevalence of side effects during the game was observed with caffeinated chewing gum, as only one participant reported hand tremors. On the contrary, previous studies have indicated that team-sport athletes habitually feel more nervousness (Pérez-López et al., 2015; Salinero et al., 2014), while the results of the present study suggest that some of the participants felt the ergogenic effects of caffeine, but not a higher level of nervousness. The low frequency of side effects observed in the present study may be explained by habitual caffeine intake of the study participants. Previous studies on team sport athletes included only light caffeine consumers (Pérez-López et al., 2015; Salinero et al., 2014), while athletes in this study were classified as mild caffeine users (Filip et al., 2020). Habitual caffeine users may develop tolerance to certain physiological effects of caffeine and thus, report the low occurrence of side effects (Filip-Stachnik, Krzysztofik, et al., 2021a; Turnbull et al., 2016), which was observed in the obtained results.

In addition to its strengths, the present study has several limitations that should be addressed: (1) the study did not include any biochemical analysis; thus, we were unable to verify the blood caffeine concentration obtained with the use of caffeinated chewing gum; (2) the study protocol included only pre- and post-game measurements, thus the impact on jump variables during the game is unclear; (3) only the effect of an acute moderate dose of caffeine (i.e., ~6 mg/kg) was analyzed and it is unknown whether different acute single or repeated doses could produce greater benefits for volleyball performance. Future studies should explore the potential benefits of using caffeinated chewing gum ingested during exercise; (4) the study sample was composed of well-trained women volleyball players, thus translating the research outcomes to men volleyball players or volleyball players with another training background should be made with caution. Finally, we assessed volleyball performance during a one-set simulated game. Further studies need to test the potential performance benefits of caffeinated chewing gums during real-life volleyball matches to simulate a more fatiguing competitive scenario.

## Conclusions

The results of the current investigation indicate that the ingestion of ~6 mg/kg of caffeine via caffeinated chewing gum significantly improves attack jump performance before and after a simulated volleyball game. Additionally, this dose and form of caffeine supplementation produces a low prevalence of side effects which are comparable to the ones produced after the ingestion of the placebo. However, the use of caffeinated chewing gum does not improve block jump height nor performance during the game, assessed by a blinded notational analysis. From a practical viewpoint, the benefits of chewing gum are somewhat minor in comparison to caffeine capsules or energy drinks, at least in this sample of mild caffeine users.
